# Excitability changes in the sciatic nerve and triceps surae muscle after spinal cord injury in mice

**DOI:** 10.1186/1749-7221-5-8

**Published:** 2010-04-18

**Authors:** Zaghloul Ahmed, Robert Freedland, Andrzej Wieraszko

**Affiliations:** 1 Department of Physical Therapy, The College of Staten Island/CUNY, 2800 Victory Boulevard, Staten Island, NY 10314, USA; 2 CSI/IBR Center for Developmental Neuroscience, The College of Staten Island/CUNY, 2800 Victory Boulevard, Staten Island, NY 10314, USA; 3 The Department of Biology, The College of Staten Island/CUNY, 2800 Victory Boulevard, Staten Island, NY 10314, USA

## Abstract

**Background:**

From the onset to the chronic phase of spinal cord injury (SCI), peripheral axons and muscles are subjected to abnormal states of activity. This starts with very intense spasms during the first instant of SCI, through a no activity flaccidity phase, to a chronic hyperactivity phase. It remains unclear how the nature of this sequence may affect the peripheral axons and muscles.

**Methods:**

We set out to investigate the changes in excitability of the sciatic nerve and to characterize the properties of muscle contractility after contusive injury of the mouse thoracic spinal cord.

**Results:**

The following changes were observed in animals after SCI: 1) The sciatic nerve compound action potential was of higher amplitudes and lower threshold, with the longer strength-duration time constant and faster conduction velocity; 2) The latency of the onset of muscle contraction of the triceps surae muscle was significantly shorter in animals with SCI; 3) The muscle twitches expressed slower rising and falling slopes, which were accompanied by prolonged contraction duration in SCI animals compared to controls.

**Conclusion:**

These findings suggest that in peripheral nerves SCI promotes hyperexcitability, which might contribute to mechanisms of spastic syndrome.

## Background

The studies of Sherrington and others showed that in chronic spinalized and decerebrated preparations reflexes were easily elicitable and responded violently to stimuli, which otherwise had no effect before injury [1,2]. Hyper-reflexia and spasticity which is velocity dependent increase in muscle tone [3], are considered as signs for corticoreticulospinal system lesions [4,5]. There is also evidence linking the development of spasticity and hyper-reflexia to changes in spinal α motor neurons excitability [6-8] spinal interneuronal hyperexcitability [9] and potentiated synaptic input with muscle stretch [10-14]. However, the exact pathophysiological mechanism which underlies muscle tone and abnormalities in reflexes is unknown. Although, there is the possibility that peripheral nerve physiology might be altered after spinal cord injury (SCI), there have been limited studies to investigate it directly. However, muscle contraction studies showed significant alteration in muscle properties after SCI [15,16] suggesting that the physiology of the peripheral axons would be altered as a result of SCI and spasticity.

A recent study by Lin et al., [17] demonstrated that the function of the peripheral nerves was altered after SCI in humans. They specifically found that peripheral nerves were of high threshold and sometimes were completely inexcitable. They attributed these results to changes in axonal structure and ion channels. However, there is always the possibility that these findings might reflect a lower motor neuron lesion in human subjects. Therefore, an investigation of axonal changes in a more controlled animal model may provide more unequivocal data.

In the present study, we asked, using an animal model, whether the nerve-muscle complex (sciatic-triceps surae) becomes hyperexcitable after spinal cord injury. We specifically hypothesized that excitability measures - amplitude, threshold, latency, conduction velocity, and stimulus-response curves of nerve and muscle - would demonstrate the characteristics of hyperexcitability in nerve-muscle complex after SCI. Moreover, we hypothesized that muscle twitches will demonstrate the properties of spastic muscle as reported by Harris et al., [15]. The present investigation gives evidence that sciatic-triceps surae complex is indeed hyperexcitable after SCI. Thus, it may provide an additional mechanism for spastic syndrome that develops after SCI.

## Methods

### Animals

Experiments were carried out on CD-1 male and female adult mice in accordance with NIH guidelines, with all protocols approved by the College of Staten Island IACUC. Animals were housed under a 12 h light-dark cycle with free access to food and water.

### Spinal cord contusion injury

Mice were deeply anaesthetized with ketamine/xylazine (90/10 mg/kg i.p.). A spinal contusion lesion was produced (n = 7) at spinal segment T13 using the MASCIS/NYU impactor [18]. The impactor was fitted with a 1 mm-diameter impact head rod (5.6 g) released from a distance of 6.25 mm onto T13 spinal cord level exposed by a T10 laminectomy. After the injury, the overlying muscle and skin was sutured, and the animals were allowed to recover under a heating lamp at 30°C. To prevent infection after the wound was sutured, a layer of ointment containing gentamicin sulfate was applied. Following surgery, animals were maintained under pre-operative conditions for ~8 months before testing. The time of recovery was selected to ensure a stable chronic SCI during testing.

### Behavioral testing

The following behavioral evaluation were performed just before the electrophysiological studies, approximately 8 months after SCI.

#### Basso mouse scale (BMS)

Motor ability of the hindlimbs was assessed by the categorical motor rating of BMS [19], using rating system of: 0, no ankle movement; 1-2, slight or extensive ankle movement; 3, plantar placing or dorsal stepping; 4, occasional plantar stepping; 5, frequent or consistent plantar stepping; no animal scored more than 5. Each mouse was observed for 4 min in an open space before a score was given.

#### Abnormal posture scale (APS)

After SCI, animals usually developed muscle tone abnormalities that were exaggerated during locomotion. We developed a posture scale to quantify the number of muscle tone abnormalities demonstrated by the animals. The rating scale ranges from 0 to 12 with a cumulative score based on the sum of the following abnormalities: limb crossing of midline, abduction, and extension or flexion of the hip joint, paws curling or fanning, knee flexion or extension, ankle dorsi or planter flexion. A score of one was given for each abnormality. The total score is the sum of abnormalities from both hindlimbs. Abnormal postures were usually accompanied by spasmodic movements of the hindlimbs.

### Electrophysiological procedures

Intact (n = 7) and SCI (n = 7) animals underwent a terminal electrophysiological experiment. Animals were anesthetized using ketamine/xylazine (90/10 mg/kg i.p). Electrophysiological procedures started approximately 45 min after the first injection to maintain anesthesia at moderate to light level [20]. As needed, anesthesia was kept at this baseline level using supplemental dosages (~5% of the original dose).

The skin covering the two hindlimbs was removed. Both triceps surae muscles were partially separated from the surrounding tissue preserving blood supply and nerves. The tendon of each of the muscles was connected to the force transducers with a hook shaped 0-3 surgical silk thread. In addition, the sciatic nerve was cleared from the surrounding tissue from the knee to the hip joint. The tissue was kept moist by drops of saline.

Both hind and fore limbs and the proximal end of the tail were rigidly fixed to the base. Muscles were attached to force displacement transducers (FT10, Grass Technologies, RI, USA); the muscle length was adjusted to obtain the strongest twitch force (optimal length). The whole setup was placed on an anti-vibration table (WPI, Sarasota, FL, USA). Animals were kept warm during the experiment with radiant heat (27°C).

**Figure 1:**
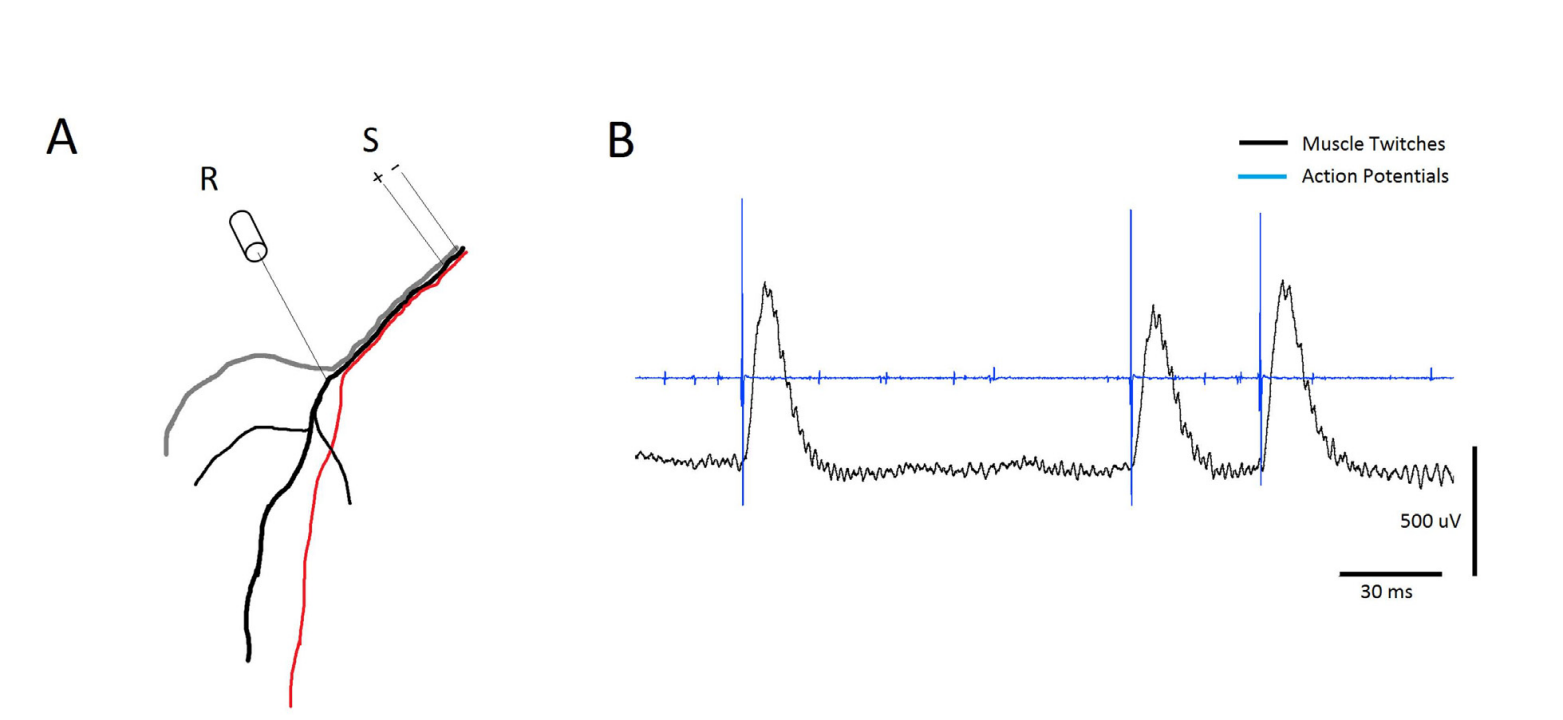
**Sciatic nerve recording**. A: anatomical illustration of sciatic nerve branches seen under the microscope. The tibial nerve (black) has three branches supplying the triceps surae muscle. The recording electrode (R) is inserted into the tibial nerve just before it enters the muscle. The stimulating electrode is situated 2 cm away from the recording electrode. B: Spontaneous activity from the tibial nerve that was correlated with muscle twitches confirming that the location of the recording (R) electrode is in the nerve bundle that innervates the triceps surae muscle. S - bipolar, stimulating electrode

A stainless steel bipolar stimulating electrode (500 μm shaft diameter; 100 μm tip; FHC, ME, USA) was set on the exposed sciatic nerve close to the hip joint (2 cm from the recording electrode) (Figure 1A). Electrode was then connected to stimulator outputs (PowerLab, ADInstruments, Inc, CO, USA). Extracellular recordings were made with pure iridium microelectrode (0.180 mm shaft diameter; 1-2 μm tip; 5.0 MΩ; WPI, Sarasota, FL, USA). The recording electrodes were inserted into the sciatic nerve branch that innervates the triceps surae muscle (Figure 1A). The proper location was confirmed by penetration-elicited motor nerve spikes, which were correlated with muscle twitches (Figure 1B). Recording electrode site was ~3 mm from the muscle. It is important to emphasize that the location of the recording and stimulating electrodes was maintained consistent across all animals. The record of extracellular activity was passed through a standard head stage, amplified, (Neuro Amp EX, ADInstruments, Inc, CO, USA) filtered (bandpass, 100 Hz to 5 KHz), digitized at 4 KHz, and stored in the computer for subsequent processing. A power lab data acquisition system and LabChart 7 software (ADInstruments, Inc, CO, USA) were used to acquire and analyze the data.

Stimulus-muscle and stimulus-nerve response curves were generated by delivering stimuli (1 ms duration), which were increased in steps (in Volts) starting from 0.05, and then increasing from 0.1, to 1.0, and from 2 to 10, in 0.1 V and 1 V increments, respectively. To determine the strength-duration time constant (SDTC), a test protocol was used with 17 stimuli of different durations (10, 20, 30, 40, 50, 70, 90, 150, 200, 300, 400, 600, 800, 1000, 2000 μs). The strength of the stimulation (mA) was adjusted accordingly for each of the durations tested to elicit minimal (all or none) triceps surae muscle response (contraction). In the same group of animals, a test stimulus of two durations (10 and 1000 μs) was used to measure the time constant of 40% of maximal muscle contraction. The threshold charge (threshold current × stimulus duration) was plotted against the stimulus duration. The time constant is given as the negative intercept of the linear regression line of the threshold charge against stimulus duration on the duration axis.

### Data analysis

F-wave was elicited by application of the stimulus equal in strength to superamaximal stimulation necessary to generate M-wave. F-wave latency was measured from stimulus artifact to the early onset of F-wave and was determined as the average of at least 10 F-waves from each animal. The peripheral motor conduction time (PMCT) was calculated by:

Where *M *is the latency of the M-response, *F *is the latency of the F-wave. The 1 ms term is a correction for the delay in re-excitation of the motoneuron [21].

We recorded the time from the start of the stimulus artifact to the onset of the first deflection of nerve compound action potential (nCAP) as well as muscle twitch. Latency of muscle twitch was also measured as the time from the earliest onset of nCAP to the earliest onset of muscle twitch. Measurements were recorded using a cursor and a time meter on LabChart software. The amplitude of sciatic nerve nCAP was measured as peak-to-peak. Analysis of muscle contractions were performed with peak analysis software (ADInstruments, Inc, CO, USA), as the height of twitch force measured relative to the baseline. Slopes for muscle contractions were extracted through Matlab-based calculations (MathWorks, Natick, MA).

### Statistical analysis

All data are reported as group means ± SEM. One sample t-tests were used for single group. Two sample student's *t*-tests (or Mann-Whitney Rank Sum Test) was used for two groups; statistical significance at the 95% confidence level. To compare multiple measurements, we performed one way ANOVA with Solm-Sidak corrections for *post hoc *analysis. Statistical analyses were performed using SigmaPlot (SPSS, Chicago, IL), Excel (Microsoft, Redwood, CA), and LabChart software (ADInstruments, Inc, CO, USA).

## Results

We used BMS and APS to identify the animals with SCI those developed locomotor and muscle tone abnormalities. BMS showed that animals with SCI had significant locomotor abnormalities (22.22% ± 4.9% of control). In addition, APS showed that animals with SCI had significant increase in the number of muscle tone abnormalities (9.1 ± 0.59).

**Figure 2:**
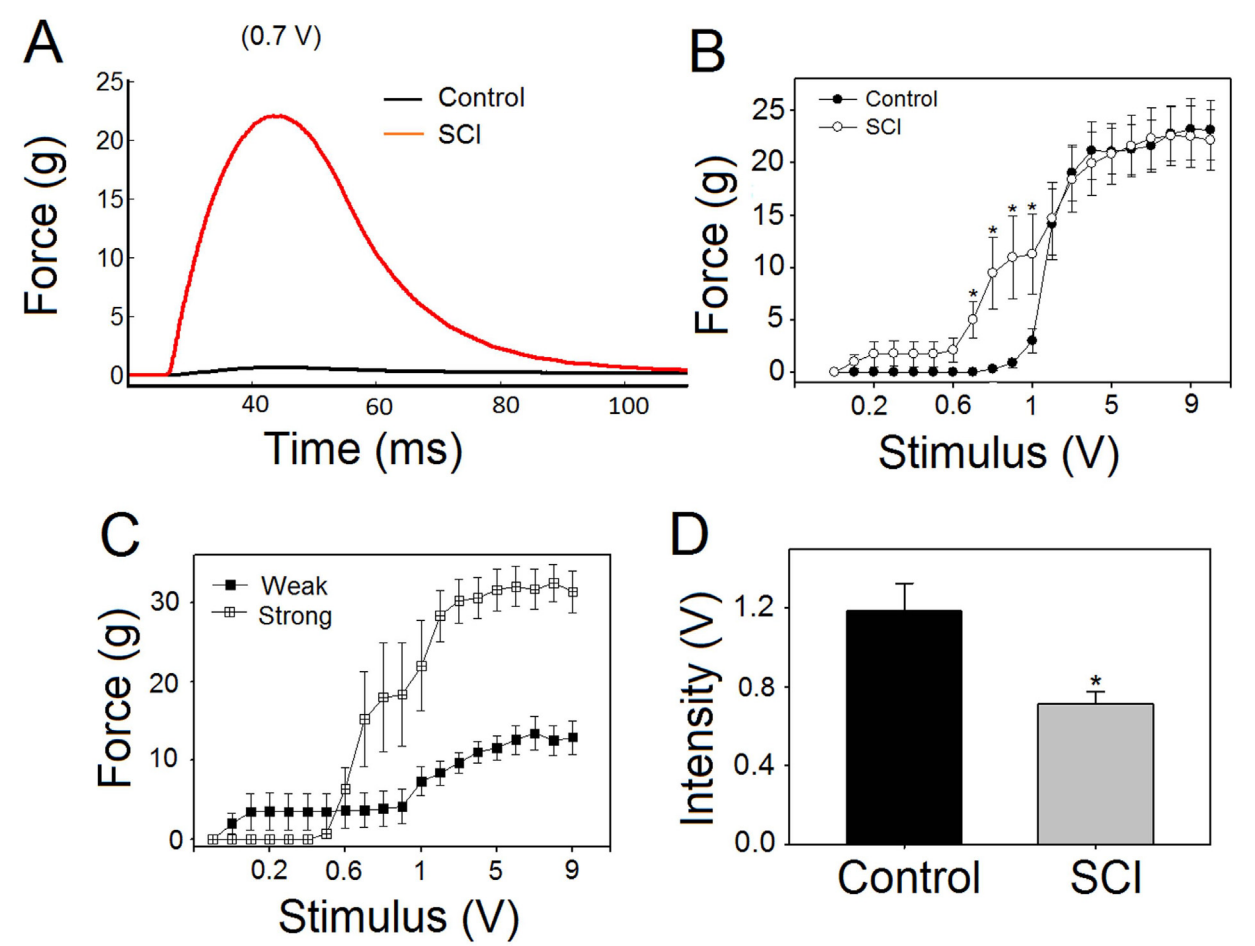
**Muscle twitches, evoked by sciatic nerve stimulation, in control and spinal cord injured (SCI) animals**. Stimulus-response curves were generated by stimulating the sciatic nerve and recording muscle twitches from the triceps surae muscles. A: Representative twitches from animals with SCI (red) and control (black) obtained by stimulus with intensity of 0.7 mA. B: Cumulative averages of the muscle responses from SCI and control animals plotted against stimulus intensity. It is noteworthy that muscle twitches are more easily evocable in animals with SCI than in controls. Note also the difference became differentiated at stimulus intensities 0.7 to 1 V (p < 0.05). C: In SCI animals, stimulus-response curve in weak muscles was different from that in strong muscles. Note that the threshold in weak muscles (0.05 V) was much lower than the threshold in strong muscles (0.6 V). D: Average minimal threshold was significantly lower in SCI animals compared to controls (p < 0.003).

To establish the excitability of the nerve-muscle complex in animals with SCI, we compared the stimulus-response (twitch force) curve generated from animals with SCI to that generated from the controls. Stimulus-response curve of SCI animals was shifted to the left at stimuli level(s) of less than 2 V (Figure 2A). This clearly suggests that nerve-muscle complex in SCI animals is of lower threshold compared to controls. The difference between SCI animals and controls was most obvious at stimulus intensities between 0.7 to 1 V (p < 0.05, Figure 2A &2B). In SCI animals, when stimulus-response curves from strong and weak muscles were compared, obvious differences emerge between the two curves (see Figure 2C). Although weak muscles had weak responses, they had very low thresholds. In SCI animals, responses from strong muscles had intermediate thresholds. The minimal threshold defined as the least stimulus intensity at which muscles would respond was also calculated. In Figure 2D, the average minimal threshold (averaged for strong and weak muscles) in SCI animals (0.71 ± 0.06 V) was significantly less than in the controls (1.19 ± 0.14 V) (p < 0.01). These results suggest that muscle contraction is more easily evocable in SCI animals, as compared to controls.

**Figure 3:**
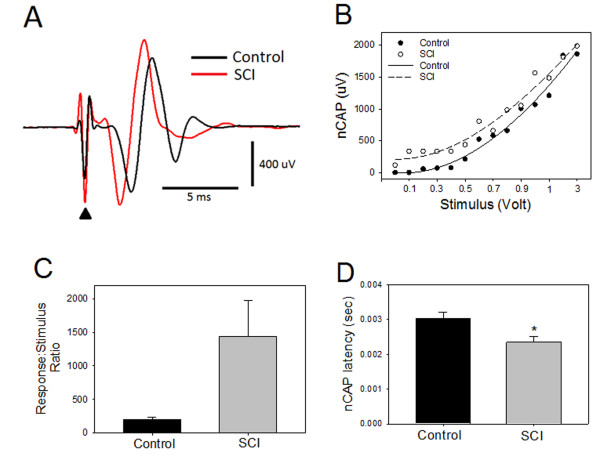
**Sciatic nerve compound action potentials (nCAP) from injured and control animals (averages for both limbs)**. A: an overlay of two nCAPs from animal with SCI (red) and control (black). Note that nCAP from SCI has shorter latency than control. Filled triangle indicates stimulus artifact. B: stimulus-response curves show that sciatic nerves responses were of low threshold and larger amplitude in SCI animals (open circles), compared to controls (filled circles). The non-linear relationship between stimulus intensity and nCAP is represented by polynomial curve fit. Note that the fit for SCI (**---**) is shifted to the left compared to controls (--) suggesting an increase in excitability. C: response:stimulus ratio was calculated for all submaximal nCAP (*p < 0.05). D: nCAP latency measured from the onset of stimulus artifact to

Since muscle contraction involves many steps before its occurrence (including nerve excitation, neuromuscular transmission and muscle membrane excitation), it should be considered an indirect measure for nerve excitability. Therefore, to estimate the nerve excitability, we recorded nCAP from the tibial branch of the sciatic nerve that supplies the triceps surae muscle. Figure 3A shows an increase in the amplitude of nCAP as well as shorter latency in nCAP recorded from SCI animals compared to controls. In Figure 3B, stimulus-response curves from SCI animals and controls show that sciatic nerve in SCI animals is of low threshold

To further illuminate this finding, the response to stimulus ratio for all submaximal nCAP was calculated and was found (Figure 3D) to be significantly higher in SCI animals (1436.3 ± 531.2%) than in controls (206.3 ± 29.7%) (p < 0.05). nCAP latency in SCI was significantly shorter (2.4 ± 0.2 ms) than in controls (3.0 ± 0.2 ms) (p < 0.01, Figure 3D).

**Figure 4:**
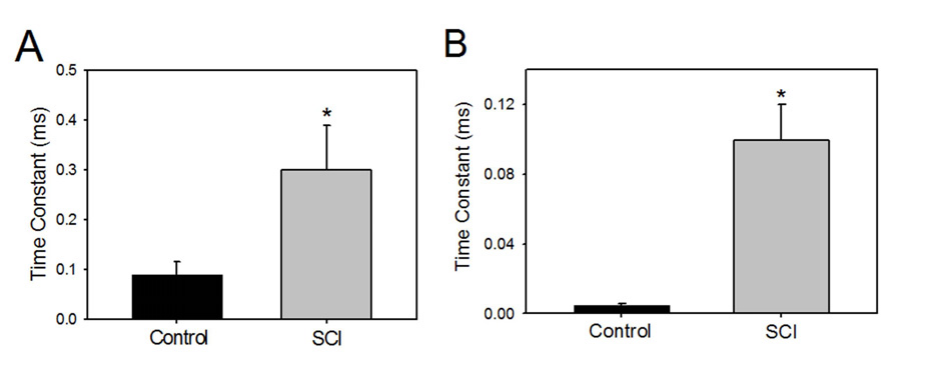
**Strength-duration time constant (SDTC) for the sciatic nerve**. A: SDTC for sciatic nerve of minimal threshold (defined as the current strength at which an all or non-response of triceps surae muscle is elicited) of control (black bar) and SCI (gray bar) animals are shown. B: SDTC was measured for the same groups of animals in A for triceps surae muscles responses of 40% of maximum (mean ± SE); stimulus durations of 0.01 ms and 1 ms were used.

SDTC was significantly higher in SCI animals (0.3 ± 0.009 ms) than in controls (0.09 ± 0.003 ms) (p < 0.02) for all or none responses (Figure 4A). SDTC was also significantly higher in SCI animals (0.1 ± 0.02 ms) than controls (0.005 ± 0.001 ms) for muscle contraction equal to 40% of maximal muscle response (p < 0.01; Figure 4B). These results suggest demyelination and/or increase in persistent Na^+ ^currents.

**Figure 5:**
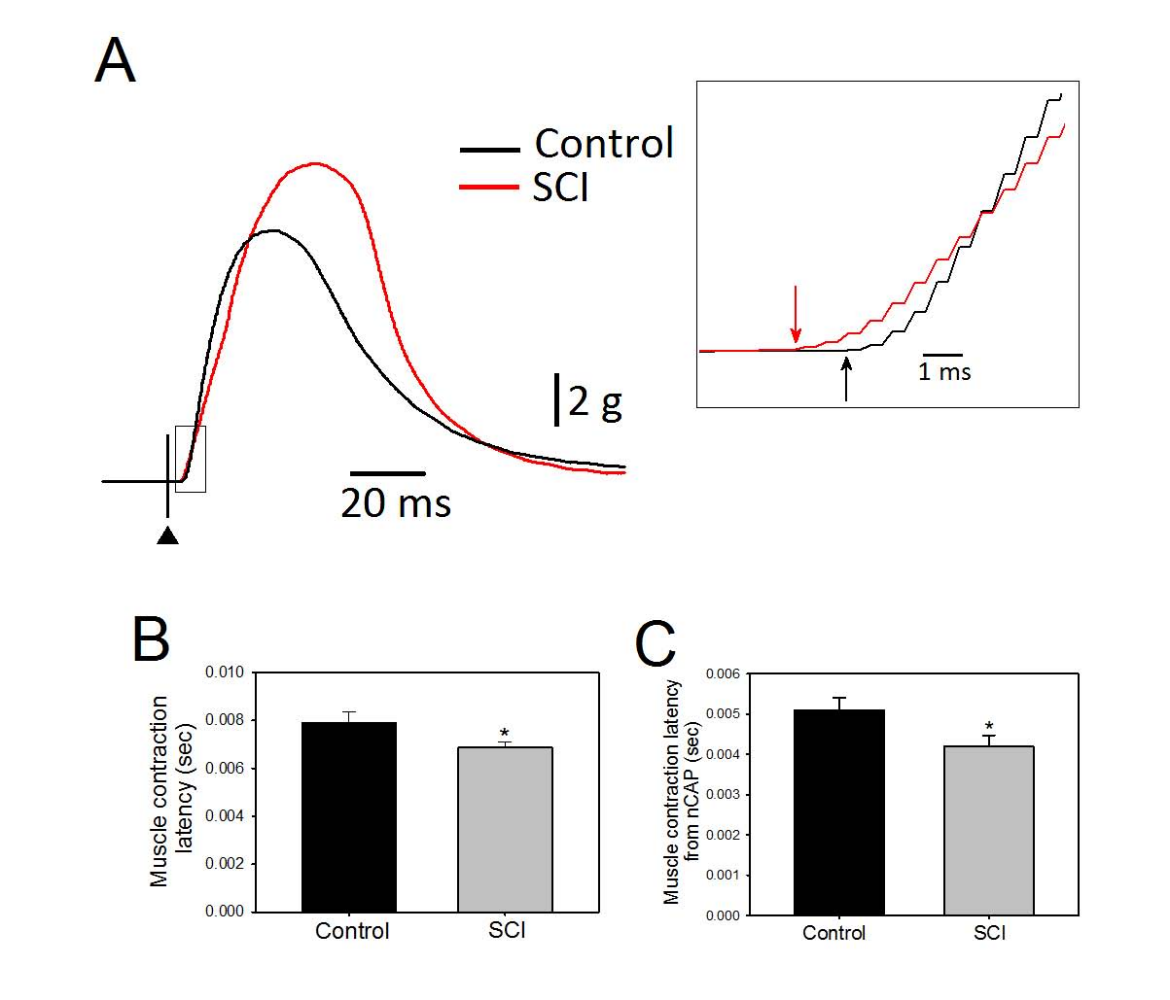
**The latency of muscle contractions**. A: an overlay of muscle twitches from control (black) and SCI animals (red). The boxed area is enlarged in the inset on the right to show the difference in the onset of muscle contraction. The filled triangle marks the time of the stimulus. B: muscle contraction latency, measured from the onset of the stimulus artifact, was significantly shorter in SCI animals compared to control (*p < 0.022). C: muscle contraction latency, measured from the onset of nCAP, was also significantly shorter in SCI animals compared to controls (*p < 0.034).

The latency of muscle contraction measured from the stimulus artifact to the onset of muscle contraction (Figure 5A), was significantly shorter in SCI animals (6.9 ± 0.3 ms) as compared to the controls (7.9 ± 0.4 ms) (p < 0.02) (Figure 5B). Latencies between the onset of nCAP and the onset of muscle contraction was significantly shorter in SCI animals (4.2 ± 0.3 ms) than in the controls (5.1 ± 0.3 ms) (p < 0.05) (Figure 5C). Similar to axons, these results indicate that muscle responses of the SCI group were rapid as well. This may be indicative of changes in either neuromuscular transmission or excitation-contraction coupling.

**Figure 6:**
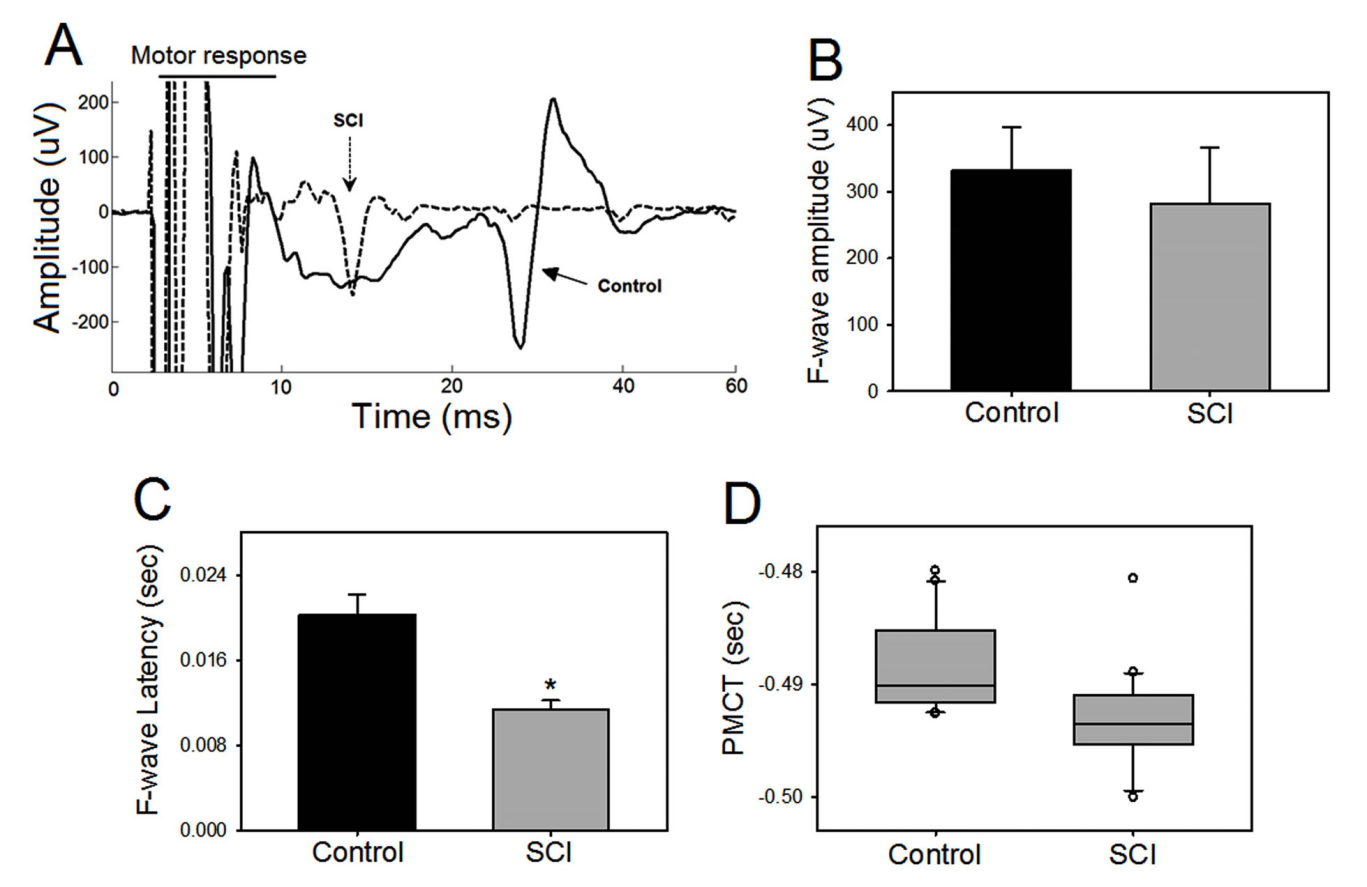
**Changes in F-wave and peripheral motor conduction time (PMCT) after spinal cord injury (SCI)**. A: superimposed traces show F-waves from control and SCI animals. Motor responses were clipped for clarity. B: there was a trend of reduction in F-wave amplitude in SCI animals however, did not reach statistical significant compared to controls (p > 0.05). C: F-wave latency was significantly shorter in SCI animals than in controls (*p < 0.001). D: PMCT was significantly lower in SCI animals compared to controls (*p = 0.001).

F-waves analyses were performed to evaluate changes along the peripheral motor nerve after SCI. Figure 6A illustrates some of the differences in F-waves between SCI and control animals. Although there was a visible reduction in the amplitude of F-wave in SCI animals, it was not statistically significant (Figure 6B). However, the latency of F-wave was significantly reduced in SCI animals (0.011 ± 0.001 sec) when compared to controls (0.021 ± 0.002 sec) (p < 0.001, Figure 6C). PMCT was also significantly reduced (-0.493 ± 0.001 sec) when compared to controls (-0.488 ± 0.001 sec) (p < 0.01, Figure 6D). Additional analysis established no significant correlation between PMCT values and measured parameters (age, body length and weight) in either SCI or control animals. Therefore we concluded that the PMCT is a good estimate for changes in conduction velocity.

**Figure 7:**
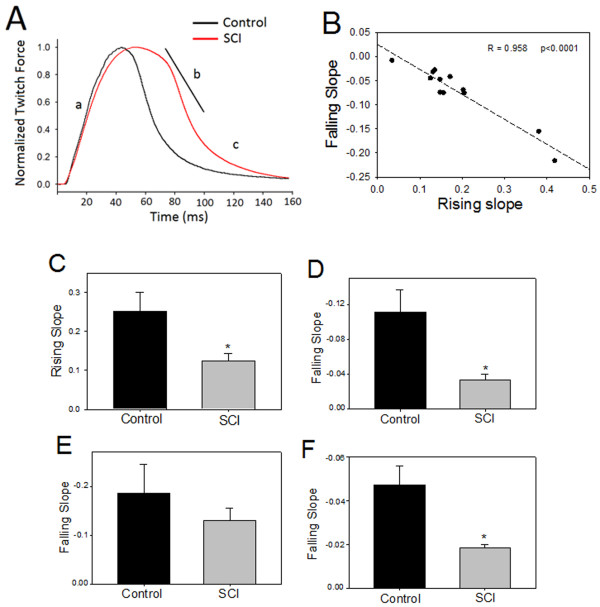
**Changes in muscle contraction properties after SCI**. A: representative normalized muscle twitches from SCI (red) and control (black) animals normalized to peak twitch force. Note the slow rising and falling of the twitch from SCI animals. The curve segments marked a, b, and c are different periods of muscle contraction that was analyzed separately below (the straight line marks the (b) segment of the curve). B: A significant correlation between rising and falling slopes, indicating that twitches with higher rising slope are associated with higher falling slope value. Data in B are from all muscles of SCI and control animals. C: The mean value of the rising slope in normal animals was significantly higher than in controls (*p = 0.033). D: The mean value of the falling slope of the entire decay period (b and c) was significantly lower in SCI animals compared to control. E: The mean value of the first part of the decay period of muscle contraction marked (b) was lower on SCI compared to control, however, the difference was not statistically significant (p = 0.411). F: The mean value of the falling slope at the last part of the decay marked (c), was significantly lower in SCI animals compared to controls (p = 0.012).

The twitch properties were analyzed to gain better understanding of the many simultaneous changes occurring in the peripheral nerves and muscles after SCI. These twitches were measured at the maximal twitch force of each muscle, using the data shown in Figure 1. Figure 7A depicts the representative twitches normalized to twitch peak force. The twitches from SCI animals appear slower in rising and falling (lower slopes) compared to the twitches from the controls. The rising and falling slopes were highly correlated (Pearson Correlation, r = 0.96, p < 0.01, Figure 7B), which indicates coupling between the processes responsible for the two events. The mean rising slope was significantly lower in SCI animals (0.13 ± 0.02) compared to those of the controls (0.25 ± 0.05) (p < 0.05, Figure 7C). The mean overall falling slope was significantly lower in SCI animals (-0.11 ± 0.03) when compared to controls (-0.03 ± 0.01) (p < 0.02, Figure 7D). The decay function of muscle twitch was divided into two periods, marked (b) and (c) in Figure 7A. Falling slopes of these two periods were calculated separately, assuming that they represent different motor units. The mean falling slope of the first period (b) in SCI animals (-0.13 ± 0.03) was not statistically significant from controls (-0.19 ± 0.06) (Figure 7E). In contrast, the mean falling slope of the second period (c) in SCI animals (-0.02 ± 0.002) was significantly lower than the controls (-0.05 ± 0.01) (p < 0.02, Figure 7F).

## Discussion

The results show that the spinal cord injury leads to increased excitability of nerve-muscle complex. Several measures of excitability were employed in the present study. An increase in nerve conduction velocity was accompanied by reduced threshold for nCAP generation and an increase in its amplitude. Thus, the muscle could be excited easier and faster. Moreover, reduction in the duration of PMCT indicates that post-injury axonal changes lead to an increase in the conduction velocity along the whole motor nerve from the spinal cord to the site of the recording electrode located very close to the muscle. These results confirm the finding in paralyzed rats by Cope et al [22], however these results contradict the finding in human with SCI [23].

Importantly, SDTC was significantly increased in the sciatic nerves of injured animals. This suggests demyelination and/or increased persistent sodium current [24]. The analysis of properties of muscle twitch in SCI animals revealed slowness in the rate of muscle contraction and relaxation. Similar changes were reported by Harris and collaborators [15] investigating segmental tail muscle in the rats. Since our experiments were performed on triceps surae muscle in mice, one can conclude that spinal cord injury might cause similar changes in all spastic muscles and across species.

In the present study, all injured animals exhibited behavioral signs of spasticity and demonstrated spasms. This indicates that the SCI that leads to spasticity may also be responsible for the increase in excitability of axons and muscles. It is known that spinal cord injury or brain damage results in hyperexcitability of neuromuscular system (expressed as dystonia, spasticity, spasm and hyper-reflexia). Although possible mechanisms of hyperexcitability may include among others the increased excitability of spinal motoneurons [6-8], spinal interneuronal hyperexcitability and potentiated synaptic input to the muscle [10-14], the exact mechanism of this phenomenon remains largely unknown. Our results expand current views on the hyperexcitability-mediating mechanisms, demonstrating that the whole neuromuscular complex becomes hyperexcitable and may participate in the mechanisms of spastic syndrome and its expression. This notion contradicts recent findings described by Lin et al., [17], who reported higher axonal threshold in human subjects with SCI. These differences may be due to a complex pathophysiology of SCI in humans which may be additionally complicated by nerve root injury. While pathophysiology of SCI in humans has been subdivided into several different types [25]which can involve both peripheral and central damage, experimental damage of the spinal cord in animals represents reproducible injury executed in a well controlled fashion. The lesions are usually localized and limited to the zone of approximately 700 μ without apparent root damage (Ahmed, unpublished observation). The effects of SCI can also depend on the type of the muscle innervated by a damaged spinal cord segment. While Lin et al., [17] evaluated motor pathway of tibialis anterior, triceps surae muscle and its innervations was the subject of our research. In support of this notion Yoshimura and Groat [26] reported that in SCI rats there was an increase in the excitability of the afferent neurons innervating urinary bladder but there was no change in neurons innervating the colon.

Inferences from the present results point to lesion-induced intrinsic changes in the peripheral axons and muscles. SDTC reflects mostly passive properties of the membrane at the nodes of Ranvier [27]. Its increase in SCI animals might indicate injury-induced demyelination and/or an increase of the expression of sodium channels (particularly persistent Na^+ ^channel) at the nodes, similarly as reported by Yoshimura and Groat [26] in afferents to urinary bladder, and observed by us in the sciatic nerve (unpublished observation). While upregulation of the sodium channel expression, or an increase in their rate activation constant [28] could reflect additional processes responsible for the increased conduction velocity, it can also be influenced by changes in axon diameter, myelin capacitance, and axoplasmic conductance [29,30]. An increase in any of these factors with the exception of myelin capacitance would increase the conduction velocity. An increase in the diameter of the spinal neurons (which enhances axoplasmic conductance) observed after SCI [31,26] could also take place in our animals and be responsible for observed increase in conduction velocity. In addition to axonal diameter, the axoplasmic conductance (and subsequently conduction velocity) can be enhanced by limited hypomyelination of the axon especially at the internode regions [32]. The hypomyelination could also induce up-regulated expression of the sodium channels and ensuing hyperexcitability, as reported for shiverer mouse brain [33].

**Figure 8:**
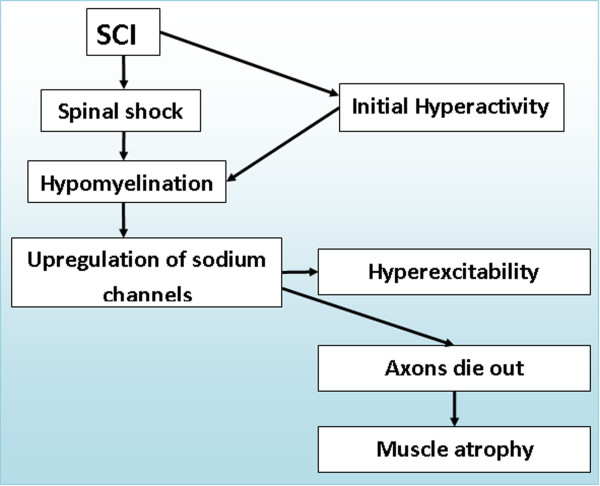
**The model of events which may lead to hyperexcitability and atrophy after SCI**. Each event may represent a potential target for clinical intervention. Two pathways are posited for hypomyelination: SCI causes immediate intense activity that may initiate mechanisms that eventually lead to hypomyelination, or the period of inactivity that followed spinal cord injury called spinal shock. Either way hypomyelination (in lesion environment) would lead to upregulation of sodium channels (Na^+ ^II) that will cause hyperexcitability. According to Waxman hypothesis [38] the activity of these channels would lead to reversed action of Na^+^- Ca^2+ ^exchanger, followed by an increase in intracellular Ca2^+^concentration, axonal death and subsequent muscle atrophy.

A model of the possible sequence of events that may lead to changes in axonal excitability is illustrated in Figure 8. The intense barrage of activity that occurs at the onset of the lesion is an event that might change the ionic composition of extra- and intra-cellular environments. As reported by us previously, the axonal excitability is regulated by its previous activity [16], and electrical stimulation of sciatic nerve causes the nerve to release preloaded glutamate analog [34]. Thus, axonal neurotransmitter release and ionic composition changes after intense activity at the onset of spinal cord lesion may play a role in the consequent axonal excitability changes. Alternatively, spinal shock that can persist up to several weeks after SCI [35], may also lead to hypomyelination followed by hyperexcitability of peripheral axons.

There was difference in threshold between week and strong muscles in animals with SCI. In that, weaker muscles expressed lower threshold than stronger muscles, becoming hyperexcitable. However, it has been reported that the weakness of the muscle induced by disuse in intact animals does not lead to hyperexcitability [36,37]. This implies that hyperexcitability is not induced by injury-related disuse of the weak and spastic muscles, but might result from interaction between the effects of disuse and lesion-induced processes.

In conclusion, we have demonstrated that the nerve-muscle complex becomes hyperexcitable in animals with SCI. The nCAP from the sciatic nerve was of higher amplitude, lower threshold, longer strength-duration time constant, and faster conduction velocity. In addition, the earliest onset of muscle contraction from the triceps surae muscle was shorter in SCI animals when compared to controls. Muscle twitches were of slower rising and falling slopes, with prolonged contraction duration in SCI animals compared to controls. These findings show that after SCI motor axons undergo excitability changes similar to their perikarya in the ventral horn of the spinal cord [6-8]. One might speculate that hyperexcitability of peripheral motor axons after SCI injury may partially underlie the expression of spastic syndrome seen after SCI.

## Competing interests

The authors declare that they have no competing interests.

## Authors' contributions

ZA design, analyze and perform the experiments and wrote the paper. RF assisted in data analysis and revising the manuscript. AW assisted in interpreting the data and in writing and revising the manuscript. All authors read and approve the final manuscript.
